# “He’s a little skinny and he’s a little wide.”: a mixed design investigation of American Indian student perceptions of healthy bodies

**DOI:** 10.1186/s12889-023-15048-5

**Published:** 2023-02-03

**Authors:** Donetta J. Cothran, Pamela Hodges Kulinna

**Affiliations:** 1 School of Public Health, Indiana University, 1025 East 7th Street, 47405 Bloomington, IN USA; 2 Arizona State University, 7271 E. Sonoran Arroyo Mall Santa Catalina Hall Rm. 330Q, 85212 Mesa, AZ USA

**Keywords:** American Indian, Children’s health, Qualitative

## Abstract

**Background:**

Childhood is a critical developmental time of wellness patterns, yet little is known about what children know and believe. Even less is known about non-majority cultures like American Indian youth. The purpose of this study was to explore American Indian students’ understandings of nutrition and physical activity.

**Methods:**

This mixed methods study took place in 10 schools in an American Indian community in the Southwestern U.S. Ninety American Indian students in grades 3–12 (8–19 years old) were interviewed. The interview included an 8-point body size chart. Numerical data were analyzed via t-test statistics while a constant comparison process and analysis was used for the interview data.

**Results:**

Students rated approximately 85% of students in Category 5 or smaller on the scale while placing 60% of adults at or above that size. There was a general trend of a larger body type for boys seen as healthy compared to that for girls. Students generally believed that their classmates were larger than the healthy body size.

For students, a healthy body was the result of compliance with “eat right and exercise” rules. They exhibited little understanding of nutrition or physical activity and there were few developmental differences in understanding. Health was a corporeal concept and violators of the eat right and exercise rules were seen as lazy.

**Conclusions:**

Students held narrow and corporeal focused notions of health focused on simple rules. People who violated the rules were “lazy”, a concept that seemed to underlie multiple constructs and a finding that holds true in other investigations. Students also reported few adult role models, a topic that should be explored with expanded family groups to better represent the multi-generational (e.g. grandparents, uncles, aunts) family housing common in the community. The findings are limited to a single American Indian community and a mixed design of relatively small numbers. This addition to the literature from a non-majority cultural group expands our knowledge of student perspectives on health. These findings can be used to create more effective curricula and interventions. Schools need more effective, but also alternately framed approaches that promote broader views of health.

## Background

Obesity levels in many countries are at epidemic levels [1] with indigenous people groups, including American Indians, having higher rates [2]. Obesity among American Indians is a major factor in health inequities across the lifestyle of Native American people. The related problems are well established with obese individuals more at risk for medical issues, [3] and psychosocial and economic challenges [4] Contributing to these trends are nutritional behaviors and food availability. Malnutrition occurs when individuals do not have enough to eat or enough of the needed nutrients. A recent scoping review of 22 studies showed Native American and Alaska Natives as reporting a weighted average prevalence of food insecurities across studies of 45.7% [5]. The other major contributing factor to these trends is low physical activity (PA). One in four adults worldwide fails to meet recommendations, while three in four of the world’s adolescents are not active at recommended levels [1] Clearly a public health need, if not a crisis, exists.

Schools have been identified as an intervention site to reverse these negative trends as schools exist in most communities, attendance is often mandatory, and health knowledge and behaviors have their roots in childhood. Understanding what students know and believe provides a strong foundation for curriculum and intervention development. Research done with young people generally finds negative attitudes toward and social problems for larger body sizes, beliefs that body size was under the individual’s control, and wishes for a different body size (e.g., Rees et al. [6, 7]). Additionally, youth’s perceptions of body size are related to physical activity with a more positive self-perception being associated with more movement [8]. The development of children’s body image is a complex issue with influences on many fronts including psychological factors, [9] family attitudes, [10] and media [11].

Children’s nutritional literacy also begins at a young age. Pre-schoolers have strong food preferences [12] and food selection takes on a moral judgment with foods identified as “good” or “bad” [13]. Even young children have ideas about the relationship between food, exercise, and weight [14]. Children’s intentions to eat healthy may not match their behavior for a variety of reasons [15]. Children’s nutrition and eating patterns are heavily influenced by family factors including food availability, modeling, mealtime structure, and family demographics like culture and socioeconomic status [16]. PA behaviors also develop in childhood with a variety of influences, including sex and weight [17].

Childhood is a key time for developing health literacy and we currently have a poor understanding of that development [18]. Without that understanding, schools are ill-prepared to serve as change agents. Schools and researchers must also understand their community as cultural differences exist in how individuals understand health (e.g., Fadiman [19]). In particular, within the American Indian community, there are distinct differences between tribes with regard to language, culture, food, and resources so a one size fits all approach to change is doomed to fail [20]. Another problem is that childhood obesity research is largely from the adult perspective and children hold unique perspectives that differ from adult views [21].

This investigation addresses these shortcomings by exploring children’s perspectives on health in a unique cultural setting. The specific research questions that guided this aspect of the project were: (1) What body types do students believe represent healthy and average students and adults? and (2) How do students explain healthy and unhealthy behaviors? This project provides critical information about children’s developmental, cultural, and personal perspectives on health.

## Methods

### Overview

The project is grounded in a constructivist framework that recognizes that students are active participants in their own education and as such, their current knowledge and beliefs are important to understand. This report is part of a larger study that examined a wellness intervention in schools that served American Indian students. Data for this paper were gathered via a single phase, concurrent nested triangulation mixed methods research design wherein the quantitative data collection was embedded within the qualitative data collection [22].

A research team consisting of university faculty members and graduate students worked closely with key school personnel and community representatives to develop the study design and data collection methods. Particular care was taken to be aware of and honor the students’ cultural norms and needs. Multiple school faculty members, and one graduate student, who were all Native American, provided feedback on the research design and materials prior to beginning. Permission to participate in the study was granted by the university, tribal council, school district, principals, individual teachers, the students’ parents, and assent was given by students. The permission process emphasized the right of the school community members to not participate, or to withdraw from participation at any time. Students could choose on a daily basis what parts of the study they would participate in or opt out of. The study design attempted to allow for additional student autonomy and comfort by allowing students to choose the interview format, whether that be a one-on-one setting or a small group of 2–3 students.

### Participants and setting

The school district was located in the Southwestern USA and served American Indian students who lived on tribal lands including 10 schools and more than 2,000 students. Participants were recruited by their classroom teachers since the larger project was a schoolwide health intervention approach. During the Fall semester, and before a school wellness intervention was fully implemented, ninety (53 female and 37 male) students were interviewed about healthy living. There were 41 elementary (grades 3 to 5, age 7–11) students, 34 middle school (grades 6 to 8, age 11–13) students, and 15 high school (grades 9 to 12, age 13–19) students.

### Data collection

**Fig. 1:**
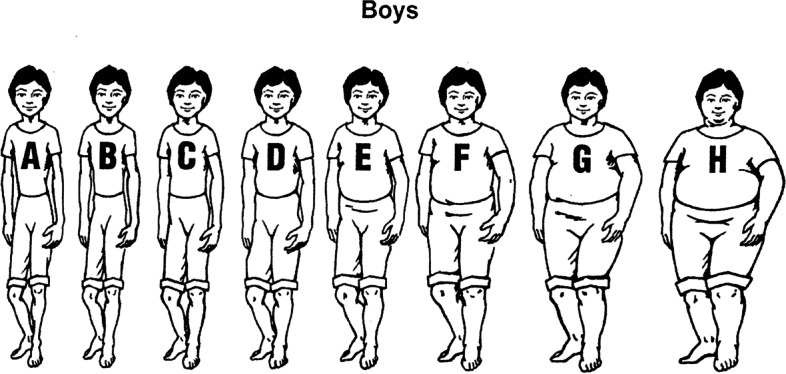
Diagram of 8 body types for boys

**Fig. 2:**
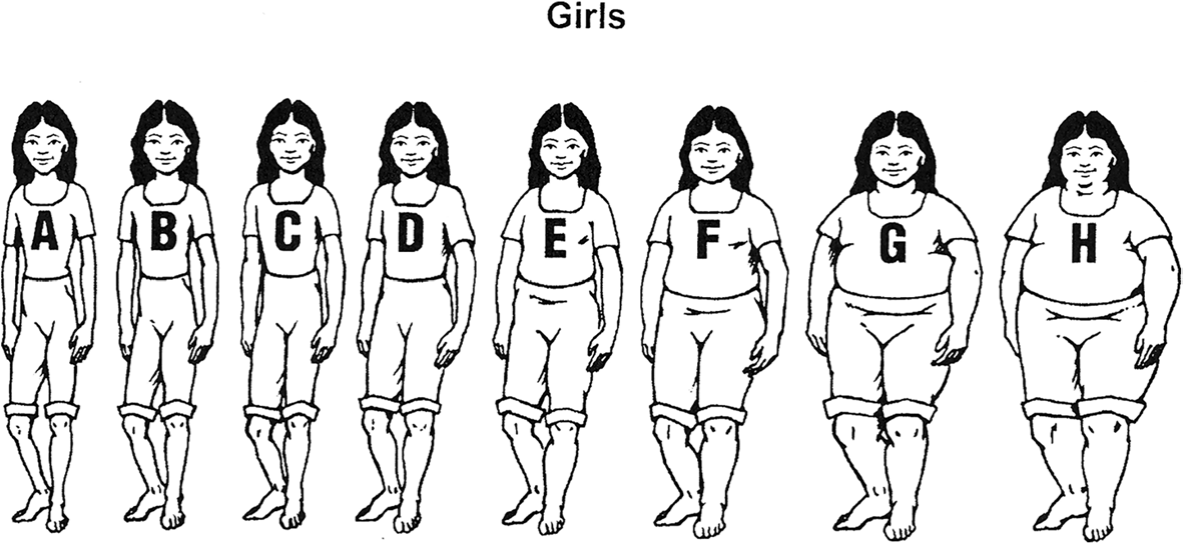
Diagram of 8 body types for girls

A semi-structured interview guide examined students’ understandings of health, with a focus on PA and nutrition. As a primary part of the interview, students were shown a diagram with eight body types ranging from thin to heavy. (Each body was labeled with a single letter from A-H). The diagrams (one for each sex; see Figs. 1 and 2) were developed for another study with American Indian children [23]. Students were asked to identify a healthy student of the same and opposite sex, and the size of “most kids” in the class for both sexes. Students also identified an average adult in their community and discussed what health might be like as an adult, but direct comparisons to an adult healthy body size were avoided to align with the students’ cultural values in which elders were deeply respected and negative talk was avoided. Students, particularly the younger ones, sometimes touched the handout to point to the body type and in that case the interviewer would state for the recording and as a check for understanding, “So you think Kid F is the healthy kid.”. Responses to the diagram prompts were probed with a specific focus on physical activity and nutrition. For example, if a student answered that body type F was a healthy body, then the interviewer would follow up with planned probes of “What do you think that kid eats?” and “What does that kid do differently from this other kid.” and the interviewer would point to a drawing opposite the one chosen by the student. If the student did not mention physical activity or nutrition, then follow up probes were used. All interviews started with a reminder that there were no right or wrong answers and if students had chosen the small group option the interviewer made clear that it was okay to give different answers because different people think differently, and all answers are right because they are your answers. Due to school schedule differences and student availability during the school day, not all students answered all questions. Fifty-five students completed the average girls in class inquiry, 78 answered which girl is healthy, 63 responded to the average boy rating, 83 answered the healthy boy question, and 68 students rated the average adult. The interviews were recorded and later transcribed.

### Data analysis

The body ratings were scored on an 8-point scale, with the thinnest drawing scored a “1” and the heaviest drawing scored an “8”. Responses and demographics were entered into a database with descriptive and *t*-test statistics analyzed using SPSS version 27. *t*-tests explored possible rating differences by sex (dichotomous) and grade (3–5 vs. 6–12).

Interview data were analyzed using inductive and iterative constant comparison methods to identify initial themes and then, as the process proceeded, common themes across participants [24]. Trustworthiness measures included triangulation of data sources from ratings, interviews, and multiple schools and grades. Peer debriefing during data collection and analysis as well as a negative case search during analysis were part of the trustworthiness protocols. Student names are pseudonyms and are followed by the student’s grade level in parentheses.

## Results

### Body diagram ratings

Table 1 presents the students’ body ratings. Students rated approximately 85% of students in Category E or smaller while placing 60% of adults at or above that size. Specific to their classmates, there was a general trend of a larger body type for boys seen as healthy compared to girls with 68.7% of students ranked type D-E as a healthy boy versus 46% ranking that size a healthy girl. Girls were more likely to be rated healthy within the A-B-C category (52.6% of students) versus boys (31.3% of students). Students generally believe their classmates to be larger than the healthy body size. *t*-test analysis for differences in ratings by group were only significantly different for *healthy girls*. Girls rated a healthy girl significantly higher (about a C) than boys (about a B) *t*(88) = 6.234, *p* = .014. Older students (grades 9–12) also rated a healthy girl significantly higher (C-D) than 3rd -5th grade students (B-C) *t*(88) = 2.273, *p* = .025.

**Table 1:** General trends of student reports of body size perceptions

Characteristics	ABC(%)	DE(%)	FGH(%)	Total(%)
Healthy boy	31.3	68.7	0.00	100
Average boy	27.0	57.1	15.9	100
Healthy Girl	52.6	46.2	1.3	100.1
Average Girl	32.7	52.7	14.5	99.9
Average Adult	4.4	35.3	60.3	100

### Body size and health

#### Student body size

In general, healthy body size was one with a just-right balance between fat and skinny. As Renita (3) explained about her healthy boy choice of D, “He’s a little skinny and he’s a little wide.” The following exchange illustrates the “just right” balance of body size concept many students espoused:

Interviewer: You both picked C as the healthy girl. What is it about that girl that made you think she was healthy?

Alyssa (5): Cause she’s skinny.

Bella (5): She’s skinny and she’s not too fat. She’s like medium.

Interviewer: So if skinny is good then why didn’t you pick A? She’s the skinniest.

Bella (5): Because she’s like too, she’s too…(Overlapping speech).

Alyssa (5): Small. She’s all small and….

Bella (5): Too very, very skinny.

Carlos (7) equated medium body size with activity in his explanation of D as the healthy boy, “He’s always exercising and he’s skinny.” When asked if being skinny was important he replied, “No. He just looks, like most kids don’t do that much, can’t do that much, but he looks active”. He offered a similar rationale for his selection of D for the healthy girl, “Because she looks like she’s been exercising a lot.” Lexi (8) chose D as the healthy body for girls because:

The other ones are skinny and they are not eating too much. The other one is just healthy. They have a little fatness on their bones. The other ones are eating too much and not getting enough exercise. So their blood pressure or something is wrong. They might get diabetes.

#### Adult body size

Students rated over 60% of adults in the F-G-H category. A small number of students, however, rated an average adult body type as an A-B-C body. When probed, students described adults with substance use disorder. Mark (7) explained, “They do drugs a lot. One doesn’t even eat because he’s always high.” Kobe (3) described an adult in the A-B range that he knew that was thin, “They smoke and drink and sometimes get high.”

### What are healthy behaviors?

Students were quick to identify a healthy body type; however, they were less sure of behaviors that led to a healthy body beyond the general guidelines of “eat right and exercise”.

They were vaguely aware of the importance of nutrition and PA but lacked specific how to or why knowledge.

#### Eating right

When Vinson (6) was asked what his healthy student does he responded, “Eat right and exercise I think.” When asked what “eat right” means he replied, “Probably eat like less junky food, probably more vegetables”. Cecilia (6), his interview partner, added some specifics, “Eating more broccoli and carrots”. “Eating right” was most often described as some combination of less junk food and more fruits and vegetables. When asked for more specifics about eating right, students often provided a food list:

Interviewer: You said the kids on this end (F-G-H) aren’t healthy. What are they doing that’s unhealthy?

Jason (3): These guys eat different like crap food and these other guys (points to A-B-C end) eat healthy food.

Interviewer: What is healthy food?

Jason (3): Carrots, bananas, vegetables, apples, grapes, and fruit.

Raegan (3): And pineapples and broccoli too.

Bryan (6) offered a list that included drinks, “They eat salad, vegetables, carrots, V8 vegetable drink, and watch what they are eating. They probably drink milk and stuff.” Sarah (8) was one of the few students to suggest a more conceptual understanding of nutrition in her response about a healthy female, “They watch what they eat. They watch their carbs. They don’t eat meat all the time.” Interestingly, she thought a healthy boy did something different, “They don’t watch their carbs. They eat whatever, but still exercise.”

#### Exercise

Exercise was the second component of the oft-given mantra of “eat right and exercise”. As with the “eat right” responses, however, students’ conceptions were limited as this exchange demonstrates:

Interviewer: You think the A girl is healthy. What would someone need to do to be healthy like her?

Raoul (3): Exercise.

Interviewer: How long should she exercise?

Raoul (3): Like four hours.

Interviewer: What kind of exercises should that person do?

Raoul (3): Running, pushups, pull-ups and sit-ups.

Many students described running/jogging and calisthenics as key aspects of being active. Advice on how often to exercise ranged from Raoul’s (3) four hours a day to Justine’s (7) run one mile per week to multiple students who answered “a lot” when asked how much exercise was healthy. By far the most common answer, however, was “I don’t know.” A few students described boys and girls doing different things:

Interviewer: Are your healthy boy and girl doing the same or different things?

Selena (8): Both are exercising.

Hannah (8): But most boys do weights.

Selena (8): Yeh.

Hannah (8): They play sports. Like they run in football and basketball and baseball.

Calbert (12) agreed that girls and boys did different things “Girls do something so that they don’t get hurt. Guys do tough stuff like football and stuff.”

#### Exercise benefits

Most students mentioned exercise as one key to being healthy, however, they did not understand why it was important. Bailey (8) and Autumn (7) were confident that being active was part of being healthy but when probed, their confusion was evident:

Interviewer: How does being active help keep you healthy?

Bailey (8): We move around a lot and we’re not, I don’t know. It just keeps your body healthy. We move our muscles.

Autumn (7): It helps us stay physically fit.

Interviewer: It helps you stay physically fit. Can you tell me what that means?

Autumn (7): No, not really.

Jose (7) shared a similar logic as he explained how exercise helped health, “It works your body. It keeps you in shape. It keeps you healthy.” When asked how exercise affects health he added, “You have movement in your body, and you get healthy.” Other students were able to give a little more detail:

Interviewer: Why is it important to exercise?

Anthony (3): I don’t know. Wait, oh, you can lose weight.

Interviewer: Any other reasons?

Anthony (3): To be strong and build bones.

Alicia (3): It just makes you healthy.

Later in the interview, Anthony (3) was asked what would happen to his body if he did not exercise and he explained “It would turn into sugar.” “Sugar” was a word used by some students as another term for diabetes. Diabetes was sometimes mentioned as a negative consequence of violating the eat right and exercise rule. When asked why it was important to exercise, Anna (7) explained, “So we all don’t get diabetes.” When asked how exercise helps with diabetes she responded, “I don’t know.” Evelyn (11) and Peter (10) knew diabetes was prevalent in the community and unhealthy, but not much more:

Evelyn (11): Diabetes like well it runs in my family. Everybody in my family has it except for the kids.

Interviewer: Is there anything you could do about that?

Evelyn (11): You have to watch what you eat and exercise and lose weight and be active.

Interviewer: How much activity should you try for?

Evelyn (11): (laughing) I don’t know.

Pete (10): It’s like watching what you are eating.

Evelyn (11): You’ve got to watch out.

Pete (10): Like for your blood pressure too.

Hannah (8) shared a similar view and noted specific benefits and consequences, “You keep your health good and can’t get diabetes very easy. It helps your heart get stronger and you keep your weight under control and strengthen your muscles.”

#### Adult behaviors

Most students believed that adults’ larger bodies occurred because they violated the eat right and exercise guidelines.

Interviewer: You told me that most of the adults you know are an H. Do you think that’s what size they were in school?

Alyssa (5): No, they were like in the A’s, B’s, C’s,

Interviewer: What do you think happened to change their body size?

Bella (5): Probably just eats too much junk food.

Alyssa (5): Yeah, she probably ate too much junk food and that’s what made her fat, like chicken and popcorn.

Bella (5): Cause that’s how my cousins are. They used to be really skinny and they got old and now they’re getting bigger and bigger.

Raoul (3) and Skye (3) agreed that adults did not eat healthy or exercise:

Interviewer: You told me most of the kids in class look like this [points to the diagram middle] but most of the adults look like this [points to the heavy end]. What do you think happened?

Raoul (3): They quit running. They quit exercising. They quit eating healthy food.

Interviewer: Why do you think they quit?

Raoul (3): They got lazy.

Skye (3): They got fat.

Interviewer: When you say they “got lazy” what does that mean exactly?

Raoul (3): They didn’t want to do anything.

Similar responses were given by Teagan (7) who explained why adults get bigger, “They get lazy. They won’t exercise or anything.” and Taylor (5), “They are probably being too lazy or something like they don’t want to do it. They probably just don’t want to do it.”

Some students were more specific in discussing challenges of the adult life to healthy behavior. Crystal (7) and Justine (7) initially explained that adults were lazy but when probed, suggested that the decreased activity was due to being old. When asked what it was about being old that kept people from moving, Crystal (7) replied, “Their bones are about to break and they work.” Her interview partner, Justine (7), added, “Yeah, they work and they don’t have time.” Pete (10) elaborated on the challenges an adult might face:

They think they got older. They just stop doing it. They just don’t have an interest in it no more so they quit. Or sometimes they get a job and a family. My uncle was that way. He used to skate and stuff and then he got a girlfriend and had a baby and then he started working. So now he doesn’t have time to do anything. He just has to think about work now.

## Discussion

Students’ health beliefs focused on the body and can be best described as a Goldilocks principle of “just right” where a body is neither too skinny nor too fat, although there was a trend to believe that girls’ healthy bodies were thinner than boys. The fact that students equated body size with health supports other student investigations where health is a corporeal concept (e.g., Burrows and Wright [25]). Most students viewed medium body types as healthy which supports some literature (e.g., Xu and Nerren [26]) and contradicted others (e.g., Montoya et al. [27]). Some of these mixed findings are methodological in nature and more consistency and better reporting is needed in future investigations [28]. More specifically the use of the diagram prompt and rating system in this study has been used with other Native American youth and the findings are similar. Stevens et al. (1999^22^) found that fourth grade Native American girls selected the body type C-D as most healthy while their male classmates thought a healthy body type was in the D-E range. Rinderknecht et al., [29] working with an age range broader than the one in this study but using the same body scale, found that body types in the mid-range (D) were reported as most healthy with younger students (some as young as 5–6 years of age) reporting that bodies smaller than Shape D were healthier. The tendency for younger students and females to have somewhat different perspectives is interesting and additional work to better understand what factors (e.g., media, gender roles, developmental influences) is needed.

Adults and their bodies were most often discussed as examples of negative health caused by “violations” of the student rules of eating right and exercise. A focus on healthy role models of various sizes would likely be helpful to students to envision their future health. Who should those role models be? Prior research has established that parents (e.g., Garriguet et al. [30]) and siblings (e.g., Kracht and Sisson [31]) influence children’s activity levels. Most of that research, however, has been conducted with nuclear family structures in Western cultures. Those models need to be expanded to include the important extended family groups (i.e., grandparents, aunts, uncles) that often exist within American Indian and other communities.

Students’ nutrition knowledge rarely extended beyond their good/bad food rule dichotomy regardless of age. A similar lack of progression across 12-to-15-year-old participants was noted by Harris et al. [32] Students’ limited PA knowledge is also a common finding (e.g., Keating et al., [33] Trost et al. [34]). Children may use descriptive norms like good/bad food rules as a decision shortcut of how to behave appropriately and those shortcuts serve as an understandable start point when young [35]. Similar simplistic descriptive norms seemed to apply to PA. Exercise was seen as sports for school-aged youth, particularly males, and exercise was walking/jogging and calisthenics for all others. The fact that few students understood more beyond those limited options is problematic for their current and future health.

In addition to limited knowledge regarding nutrition and PA, students also held limited conceptions of health in general. When asked what a healthy person does, students almost never mentioned social, emotional, or spiritual topics related to health and instead spoke exclusively about the physical domain. A particularly important omission to note was students’ lack of discussion about enjoyment and PA. Enjoyment is key to children’s engagement in PA (e.g., Lewis et al., [36] Pearce et al. [37]). Understanding youth PA also means recognizing the cultural and social capital youth associate with movement (e.g., Everley and Macfadyen [38]; Jago et al. [39]). For example, students are more influenced by the social impact of being fat than the health risk aspects (Everley and Everley; [40] Rees et al. [6, 7]). Perhaps the students’ limited view is not surprising given that schools have been criticized for offering a narrow view of health in which:

…little attention is paid to other constructions of health that may be more meaningful to cultural groups other than the white middle-class, nor to broader notions of health which would prioritise other aspects of health such as connectedness to community, respect for elders over a preoccupation with personal health issues [41] (p224).

This culturally narrow, limited view is a critical factor in students’ (dis)engagement, particularly those from the non-majority culture.

Given these findings and the call for schools to serve as intervention sites to promote better health for students, what should schools do? An obvious first step is to create deeper and differentiated health curricula that address the different needs of different aged students and allows them to grow their knowledge and experiences over time. Those curricula must be culturally sensitive to local communities and people groups. Beyond just cultural sensitivity, the curricula could approach food and movement from a more critical and historical perspective to help students understand their peoples’ diets prior to colonization and to connect those past and current food patterns with their culture and language (e.g. Decolonizing Diet Project [42]) That reframing would also help address some of the other problematic issues inherent in the adoption of a Western diet and the many changes that have occurred in Native American life in the last century [43]. Another step suggested by Russell-Mayhew and Grace [44] is to reframe current programs which focus on risk avoidance instead of health promotion. They suggest programs should not equate weight/BMI with health and should help students develop critical thinking skills to better understand the complexities and inequalities of health and change agents to those patterns. Schools alone, however, are unlikely to be successful in addressing such complex topics and instead they should be part of multi-dimensional approaches that include families, community, and health services that focus on the community assets as well as needs. Culturally sensitive collaborative approaches like the Pathways to Health program which used tribal health ambassadors and culturally appropriate talking circles as a means of data collection and communication hold promise, [45] but much more work is needed to identify a variety of effective tools to meet the needs of different people groups and contexts.

This study successfully explored an at-risk and underrepresented student population. This is important because youth are different from adults and cultural perspectives on health vary. Students have clearly learned the initial messaging of a healthy lifestyle and adopted descriptive norms of eating right and exercise, but their knowledge base never developed beyond that point and their body size-focused health interpretations and narrow views of health were problematic for developing healthy lifestyles. These findings can guide curricula and interventions. Using the diagram to prompt student conversation and triangulate data is another positive component of the overall design. Allowing students to choose to be part of a small group for interviews allowed the research team to increase student comfort and access more student perspectives, however, it is possible that students felt peer influence on their answers. Although the inclusion of American Indian students is a strength of the design, the fact that only one tribe was involved does limit the findings to that group. The sample also included more young students than older students and very young students (K-2) were not involved at all. Future research should broaden across other cultural groups and ages. Finally, the unpredictable nature of the school day meant that not all students were able to answer all questions as scheduled interviews were cancelled or shortened regularly which is less than ideal.

## Conclusion

These findings provide insights into American Indian children’s perspectives on health and those findings can be used to create more effective curricula and intervention efforts. It is clear that students know diet and exercise are important, but their limited eat right and exercise mantra provides few actual life skills and health paths to consider. It is also clear that broader views of health with less focus on body size and more focus on total wellness and the enjoyment that health can bring are also needed in school and community programming.

## Data Availability

The data that support the findings of this study are available on request from the corresponding author [DC]. The data are not publicly available due to the formal agreement with the American Indian tribe involved to maintain strictest privacy and non-identification of tribal members as well as specific events and locations.

## Abbreviations

PA: Physical Activity
WHO: World Health Organization
